# Artificial intelligence in medical education: Performance of ChatGPT versus medical students on medical examination

**DOI:** 10.1097/MD.0000000000050323

**Published:** 2026-08-21

**Authors:** Kyung Wook Kim, Hong Bae Jeon, Jong Hyuk Choi, Dong Uk Lee, Yoon Sung Jeon, Bum-Jin Shim

**Affiliations:** a Department of Orthopaedic Surgery, Dankook University Hospital, Dankook University College of Medicine, Cheonan, South Korea; b Department of Plastic and Reconstructive Surgery, Dankook University Hospital, Dankook University College of Medicine, Cheonan, South Korea; c Department of Preventive Medicine, College of Medicine, Dankook University, Cheonan, South Korea; d Research Institute of Healthcare Bigdata, College of Medicine, Dankook University, Cheonan, South Korea; e Department of Orthopedic Surgery, Yeungnam University Hospital, Yeungnam University College of Medicine, Daegu, South Korea.

**Keywords:** artificial intelligence, ChatGPT, examinations, large language models, medical education, medical students, multiple-choice question

## Abstract

Attention toward Chat Generative Pre-trained Transformer (ChatGPT) has greatly increased, including in the medical field, but concerns about its problem-solving ability, accuracy, reliability, and limitations in medical problems always coexist. This study aimed to evaluate ChatGPT’s performance in solving a medical school examination. We used 50-question examinations from the preventive medicine and psychiatry fields applied to 41 fourth-year medical students. Forty-nine questions were separately entered (one was excluded for being image-based) into the text box of ChatGPT models 3.5 and 4.0 with a request to select the single best answer. The initial answer was considered for primary evaluation, but incorrect answers led to model re-prompting to evaluate its answer regeneration function. We assessed the accuracy of both ChatGPT models and compared them with that of students. The student mean score was 29.5 ± 5.9. ChatGPT’s performance was higher in model 4.0 and when re-prompted (models 3.5 and 4.0 initial scores, 28 and 43; re-prompt scores, 40 and 46). Model 4.0 outperformed all students at re-prompting. Both ChatGPT models and all prompts (except for the model 3.5 first prompt) had a higher probability than students of providing correct answers. For easy questions, the accuracy of both ChatGPT models differed significantly from that of students. For difficult questions, the difference between students and the first prompt of model 3.5 was nonsignificant. We observed a nonsignificant tendency in the point estimation when comparing the accuracy of ChatGPT models and prompts by question difficulty. Both models showed excellent performance and learning ability in the medical field, especially model 4.0, and can be considered reliable large language model tools. However, future studies should constantly verify the reliability of the information from these models. Also, educators must be cautious when interpreting/applying ChatGPT output data for clinical and educational purposes.

## 1. Introduction

Over the recent decades, advances in artificial intelligence (AI) have changed various human tasks and their respective fields. Among the various advances, large language models (LLMs) have recently taken the spotlight through their use of deep learning.^[1]^ LLMs are trained on large amounts of data and use self-supervised learning, repeating the training and learning process several times until achieving an acceptable accuracy level. This enables LLMs to generate text, answer questions interactively, and translate or classify text.^[2–4]^

Among the various LLM types, there is the powerful Chat Generative Pre-trained Transformer (ChatGPT) chatbot developed by OpenAI.^[3]^ Its growth has been rapid, leading it to become a hot topic in several fields. ChatGPT can simulate a conversation as if it were a human and provide consistent explanations on various topics, including medical departments.^[2,5–7]^ This renders it useful as a complementary tool for education and increases people’s easy access to medical information. With the great interest placed on ChatGPT worldwide, various studies have been conducted regarding its capability in the medical field.^[8–11]^ However, research specifically evaluating LLM accuracy in solving medical problems remains lacking, and there are honest concerns about LLMs’ knowledge limitations regarding specialized medical fields and the potential delivery of incorrect information for users.

There are also 2 versions of ChatGPT, namely model 3.5 (i.e., the free version) and advanced model 4.0 (i.e., the paid version, which has improved accuracy and processing capabilities), and comparative evaluations of these versions are necessary. Lee et al showed that ChatGPT model 4.0 demonstrated better performance than ChatGPT model 3.5 in answering a random selection of emergency medicine board examination questions in the Korean language.^[12]^ Studies on these topics hold potential to clarify the limitations, accuracy, and reliability of ChatGPT, which may be useful as a reference tool for all those professionals who deliver medical information.

This study thus attempted to evaluate the performance of ChatGPT on a medical school student multiple-choice question examination. We aimed to determine the following 3 major points: how many correct answers ChatGPT can provide to the questions in the medical school student examination; how it fares against the performance of medical students in the examination; and the accuracy difference between ChatGPT models 3.5 and 4.0.

## 2. Materials and methods

### 2.1. Ethics

This study’s design was approved by the Institutional Review Board of the Dankook University College of Medicine/Dankook University Hospital (no. 2024-01-006), which waived the need for informed consent owing to its retrospective nature.

### 2.2. Input data

The examination of interest was the final multiple-choice question examination for the fields of preventive medicine and psychiatry applied to fourth-year medical school students on August 23, 2023. The questions in the examination were validated by a board-certified professor in the Preventive Medicine and Psychiatry Department of the Dankook University College of Medicine on January 8, 2024 and there were no errors or revisions in the questions. Therefore, our input data represented true out-of-training samples for the Generative Pre-trained Transformer models. Forty-one students completed the test, which comprised 50 questions (49 text-only questions and 1 image-based question), each with 5 response options and only 1 correct answer.

Among the various LLM types, we used ChatGPT models 3.5 and 4.0. Among the questions, only the single image-based question was ruled out of the study because it could not be applied to the ChatGPT models. This means that we assessed ChatGPT’s abilities using 49 questions, which in turn were separately entered into the text box of ChatGPT along with a request for it to select the single best answer. To reduce memory retention bias, a new chat session was administered for each question. While we considered only ChatGPT’s initial answers for primary evaluation, when the derived answer was incorrect, we asked the question again (i.e., re-prompt) to evaluate its response regeneration function. This process was performed using both ChatGPT models 3.5 and 4.0.

Finally, we assessed the accuracy of the 2 different ChatGPT models on the first prompt and the re-prompt and compared their performances with those of medical students. We used a private mode of ChatGPT to prevent others from accessing our records.

### 2.3. Adjudication and statistical analysis

Each question scored 1 point, and the percentage of students who responded correctly to each question was calculated. In addition, the mean values of the students’ scores were evaluated. McNemar test was used to compare the accuracy of the ChatGPT models for paired questions, and *t* test was used to compare the accuracy of students with that of each ChatGPT model and their prompts. Differences in performance across the ChatGPT models and students were further analyzed while stratifying the questions into difficult and easy ones (i.e., based on students’ median accuracy; difficult questions: greater than or equal to the median; easy questions: less than the median). We used the chi-square test and Fisher exact test to evaluate performance differences across ChatGPT models and prompts based on question difficulty. Statistical significance was set at *P* < .05, and all statistical analyses were performed using R version 4.3.1 (R Foundation for Statistical Computing).

## 3. Results

### 3.1. Comparison of the report card of ChatGPT with that of medical school students

**Figure 1.**
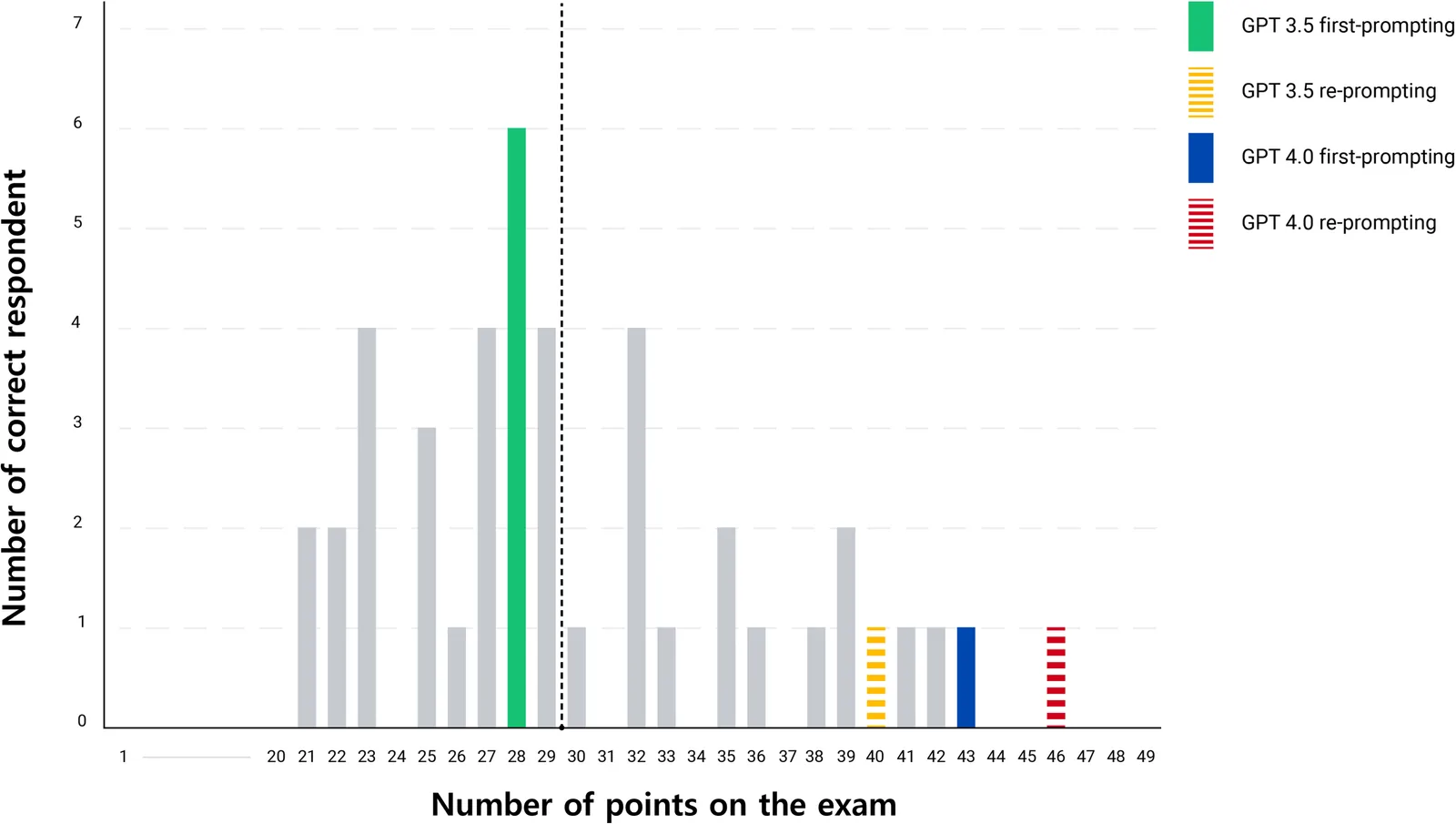
Distribution of scores on the examination. The figure shows the number of medical students who answered each of the 49 test questions correctly. The average score for students who took the test was 29.5 (dotted line), and the average score for the first prompt of ChatGPT model 3.5 was 28 which is below the average of students. The dotted box represents cases where there are no students and only the correct answers from the GPT are available. ChatGPT = Chat Generative Pre-trained Transformer, GPT = Generative Pre-trained Transformer.

Based on the report card of medical students, the mean score was 29.5 ± 5.9 (Fig. 1). Of the 41 students, there was 1 student who took first place (43 points) and 1 other who took second place (42 points). Regarding the performance of ChatGPT, model 3.5 scored 28 points (out of 49 questions) at the first prompt, and 6 students scored 28 points, which was lower than the mean score of students. However, at re-prompt, model 3.5 scored 40 points, which would equate to fourth place when comparing with the ranking of students. ChatGPT model 4.0 scored 43 points at the first prompt, and 1 student scored 43 points, thus being equivalent to first place when considering student ranking. The re-prompt of model 4.0 led to a score of 46 points, a result 16 points higher than the average score of medical students, and 3 points over in relation to the first place.

### 3.2. Percentage of correct answers: medical students versus ChatGPT models 3.5 and 4.0

**Figure 2.**
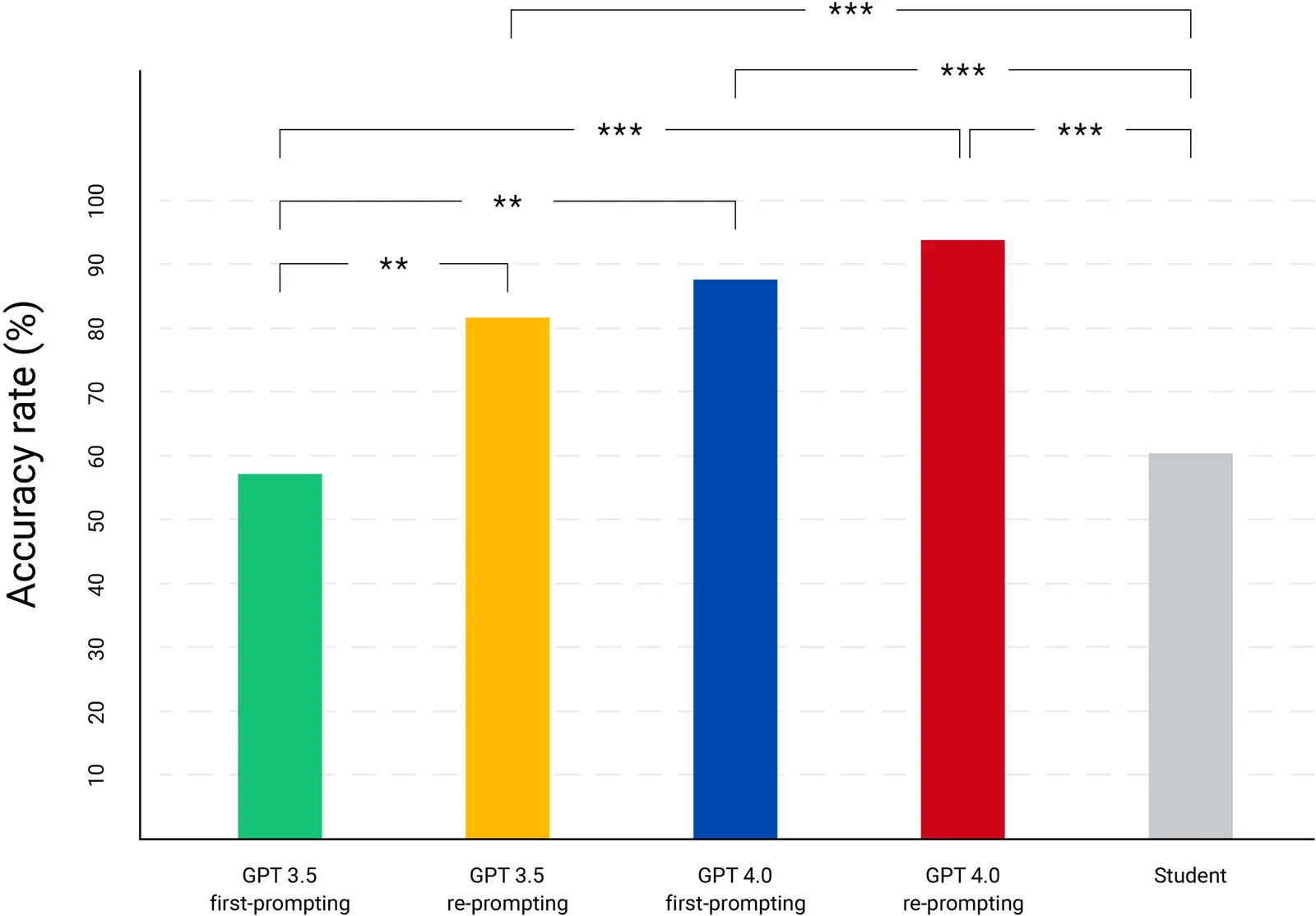
Comparison of accuracy rates between ChatGPT models, prompts, and students. “**” indicates statistical significance (*P* < .001) and “***” indicates statistical significance (*P* < .0001). ChatGPT = Chat Generative Pre-trained Transformer, GPT = Generative Pre-trained Transformer.

Upon converting the scores obtained by the ChatGPT models and students into percentages, the first prompt and the re-prompt of ChatGPT model 3.5, the first prompt and the re-prompt of ChatGPT model 4.0, and students showed the following values: 57.1%, 81.6%, 87.8%, 93.9%, and 60.2%, respectively (Fig. 2). The accuracy rates of the remaining ChatGPT model and prompts showed a significantly different distribution from that of the first prompt of model 3.5. However, there were no significant differences in distributions between the re-prompt of model 3.5 and the first prompt of model 4.0, the re-prompt of model 3.5 and that of model 4.0, and the first prompt and the re-prompt of model 4.0 (Table 1). In addition, when comparing the accuracy rates between ChatGPT models and students, significant differences were confirmed between students and all ChatGPT models and prompts, except for the first prompt of model 3.5 (Table 2).

**Table 1:** Comparison of accuracy between 2 versions of ChatGPT.

	Total number of questions (N = 49)			Easy questions (N = 25)			Difficult questions (N = 24)		
	GPT 3.5 first prompting		*P* value	GPT 3.5 first prompting		*P* value	GPT 3.5 first prompting		*P* value
GPT 3.5 re-prompting	Correct	Wrong		Correct	Wrong		Correct	Wrong	
Correct	28	12	*.001*	17	6	*.041*	11	6	*.041*
Wrong	0	9		0	2		0	7	
	GPT 3.5 first prompting			GPT 3.5 first prompting			GPT 3.5 first prompting		
GPT 4.0 first prompting	Correct	Wrong		Correct	Wrong		Correct	Wrong	
Correct	25	18	*.002*	17	7	*.023*	8	11	.061
Wrong	3	3		0	1		3	2	
	GPT 3.5 first prompting			GPT 3.5 first prompting			GPT 3.5 first prompting		
GPT 4.0 re-prompting	Correct	Wrong		Correct	Wrong		Correct	Wrong	
Correct	27	19	*<.001*	17	8	*.013*	10	11	*.009*
Wrong	1	2		0	0		1	2	
	GPT 3.5 re-prompting			GPT 3.5 re-prompting			GPT 3.5 re-prompting		
GPT 4.0 first prompting	Correct	Wrong		Correct	Wrong		Correct	Wrong	
Correct	36	7	.547	22	2	1.000	14	5	.724
Wrong	4	2		1	0		3	2	
	GPT 3.5 re-prompting			GPT 3.5 re-prompting			GPT 3.5 re-prompting		
GPT 4.0 re-prompting	Correct	Wrong		Correct	Wrong		Correct	Wrong	
Correct	39	7	.077	23	2	.480	16	5	.221
Wrong	1	2		0	0		1	2	
	GPT 4.0 first prompting			GPT 4.0 first prompting			GPT 4.0 first prompting		
GPT 4.0 re-prompting	Correct	Wrong		Correct	Wrong		Correct	Wrong	
Correct	43	3	.248	24	1	1.000	19	2	.480
Wrong	0	3		0	0		0	3	

Italic *P* values indicate statistically significant differences (*P* < .05).

ChatGPT = Chat Generative Pre-trained Transformer, GPT = Generative Pre-trained Transformer.

**Table 2:** Comparison of accuracy between the student group and the ChatGPT models.

N (%)	Total number of questions			Easy questions			Difficult questions		
	49 (100)			25 (100)			24 (100)		
	μ	Mean of students’	*P* value	μ	Mean of students’	*P* value	μ	Mean of students’	*P* value
ChatGPT 3.5									
First prompting (%)	57.1	60.2	.110	68.0	80.4	*<.001*	45.8	42.4	.243
Re-prompting (%)	81.6	60.2	*<.001*	92.0	80.4	*<.001*	70.8	42.4	*<.001*
ChatGPT 4.0									
First prompting (%)	87.8	60.2	*<.001*	96.0	80.4	*<.001*	79.2	42.4	*<.001*
Re-prompting (%)	93.9	60.2	*<.001*	100.0	80.4	*<.001*	87.5	42.4	*<.001*

Italic *P* values indicate statistically significant differences (*P* < .05).

ChatGPT = Chat Generative Pre-trained Transformer.

**Figure 3.**
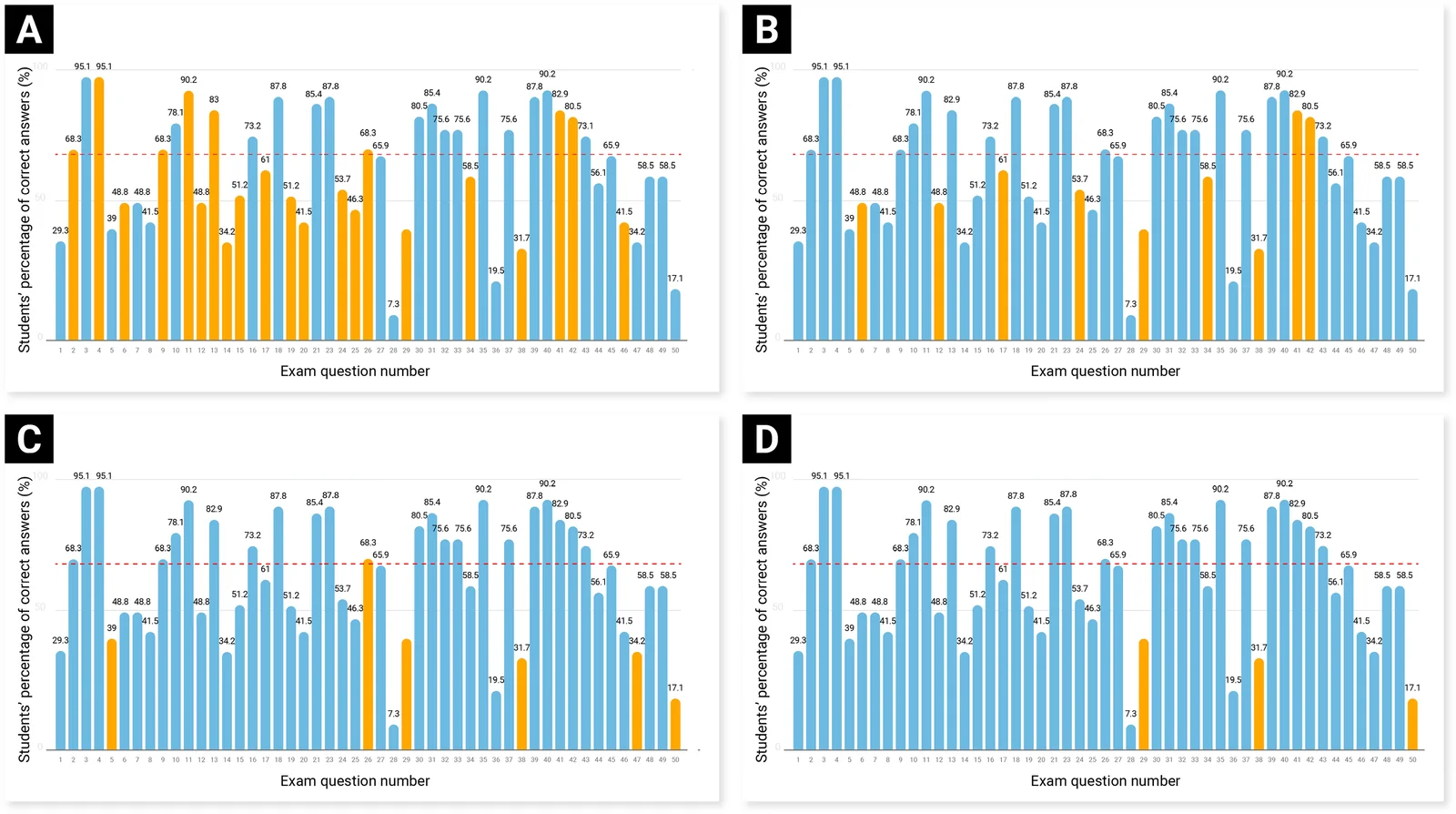
Distribution of correct responses of the ChatGPT models and prompts in relation to the students’ percentage of correct answers. For the difficult questions, there was a nonsignificant difference only between students and the first prompt of model 3.5 (red dotted lines show medians. Question 22 was not included in the analysis because it was an image-based question). Blue bars indicate correct answers, orange bars indicate incorrect answers (A, GPT 3.5 first-prompting; B, GPT 3.5 re-prompting; C, GPT 4.0 first-prompting; D, GPT 4.0 re-prompting). ChatGPT = Chat Generative Pre-trained Transformer.

Figure 3 shows that the performance of ChatGPT improved from models 3.5 to 4.0, and from the first prompt to the re-prompt. As aforementioned, we stratified the examination questions into 2 groups (easy and difficult questions) based on the median (65.9%) of students’ correct responses to the entire examination (question 22 was excluded for being image-based). The easy questions were 2, 3, 4, 9, 10, 11, 13, 16, 18, 21, 23, 26, 27, 30, 31, 32, 33, 35, 37, 39, 40, 41, 42, 43, and 45, while the difficult ones were 1, 5, 6, 7, 8, 12, 14, 15, 17, 19, 20, 24, 25, 28, 29, 34, 36, 38, 44, 46, 47, 48, 49, and 50. There were significant differences in accuracy rates for easy questions between the first prompt and the re-prompt of model 3.5, between the first prompt of model 3.5 and that of model 4.0, and between the first prompt of model 3.5 and the re-prompt of model 4.0. Moreover, there were significant differences in the accuracy rates for difficult questions between the first prompt and the re-prompt of model 3.5, and between the first prompt of model 3.5 and the re-prompt of model 4.0. However, no differences were observed among the remaining model prompts (Table 1).

When comparing the accuracy rates for easy and difficult questions in each ChatGPT model, the first prompt of model 3.5 had an accuracy rate of 45.8% for difficult questions, while the re-prompt of model 4.0 showed an accuracy rate of 100% for easy questions. Otherwise, when comparing the accuracy rates between students and all prompts of the 2 ChatGPT models, all model prompts differed significantly from students for easy questions, but there was no significant difference between students and the first prompt of model 3.5 for difficult questions (Table 2). We then checked whether the accuracy of the ChatGPT models varied depending on question difficulty. Although we found a tendency in the point estimation, we could not confirm statistical significance (Table 3).

**Table 3:** Comparison of accuracy between the ChatGPT models based on question difficulty.

	Total number of questions	Easy questions	Difficult questions	*P* value
	49 (100)	25 (100)	24 (100)	
ChatGPT 3.5 first prompting				.201
Correct	28 (57.1)	17 (68.0)	11 (45.8)	
Wrong	21 (42.9)	8 (32.0)	13 (54.2)	
ChatGPT 3.5 re-prompting				.074
Correct	40 (81.6)	23 (92.0)	17 (70.8)	
Wrong	9 (18.4)	2 (8.0)	7 (29.2)	
ChatGPT 4.0 first prompting				.098
Correct	43 (87.8)	24 (96.0)	19 (79.2)	
Wrong	6 (12.2)	1 (4.0)	5 (20.8)	
ChatGPT 4.0 re-prompting				.110
Correct	46 (93.9)	25 (100.0)	21 (87.5)	
Wrong	3 (6.1)	0 (0)	3 (12.5)	

Values are presented as number (%).

ChatGPT = Chat Generative Pre-trained Transformer.

## 4. Discussion

In the current study, we showed that the performance of ChatGPT exceeded the average score of medical students in the medical school examination. The ChatGPT model 4.0 showed a particularly outstanding performance, being better than that of the medical student with the highest score. The findings also show ChatGPT’s excellent learning ability based on its higher scores at re-prompt than at the first prompt.

Since its launch in November 2022, ChatGPT has been in the spotlight worldwide because of its demonstration of the usefulness of AI in generating responses based on input data, representing a step toward opening up a new era in human history. In particular, ChatGPT can be used for question-answering, translation, and can be applied in various medical fields,^[2]^ and its public nature has enabled ChatGPT users worldwide to easily obtain medical information. In fact, recent years have seen several studies depict the application and usefulness of ChatGPT in medical research and in the processing of medical data.^[5–7,12,13]^ In most countries worldwide, people evaluate and objectivize the accuracy of the professional knowledge of a person through licensing examinations that yield professional certifications and qualifications. No wonder there have been studies in recent years that have investigated how ChatGPT would fare in some professional licensing examinations. For example, Kung et al^[11]^ showed that ChatGPT passed the US medical licensing examination, while Kim et al^[14]^ reported the excellent performance of ChatGPT in solving the questions in an orthopedic board certification examination.

However, concerns remain regarding ChatGPT’s accuracy and reliability. Specifically, the answers generated by ChatGPT sound like “obvious information” that should be accepted as fact, but there have been instances where it afforded users wrong answers or where there have been changes in responses. These issues have raised concerns among users worldwide in light of the potential impact of ChatGPT, particularly in medical fields.^[7]^ Thus, although the competency of ChatGPT is regularly evaluated, it can still deliver incorrect information, which can, in turn, cause harm to people receiving the information. This makes it crucial to evaluate the accuracy of ChatGPT and verify its application in medical fields.^[15]^ Our study shows that although ChatGPT may contain incorrect information, it can be considered a reliable LLM tool.^[2]^ It is, notwithstanding, not perfect and needs to be constantly verified to ensure reliable information provision, and users must be cautious when interpreting the output data.

Regarding accuracy, the ChatGPT model 4.0 outperformed model 3.5 in overall accuracy for both the first prompt and the re-prompt, showing excellent outcomes in our study. There have been reports that ChatGPT is vulnerable to tests that include media.^[5]^ Although media was excluded from our study, the results for ChatGPT model 4.0 appeared to be superior to those for ChatGPT model 3.5. For model 3.5, the re-prompt outcomes were above average in comparison to those of students, whereas its first prompt results were below the average scores of students. ChatGPT model 3.5 has been known to struggle with higher-order and longer questions,^[16,17]^ and we accordingly believe that this model of the LLM is not well equipped for tackling medical school examinations and is not as capable as ChatGPT model 4.0. A study by Lee et al demonstrated the ability of ChatGPT in solving the Korean emergency medicine examination, and ChatGPT model 4.0 was superior to model 3.5.^[12]^ However, because various studies report that ChatGPT model 3.5 passed the board certification examination in several subspecialized departments, further verification is needed.^[11,14]^

In the current study, the re-prompt accuracy showed a statistically significant increase compared with the first prompt, regardless of the ChatGPT model. Few previous studies have shown similar results.^[18–20]^ This demonstrates the excellent learning ability of ChatGPT models and reflects the potential of the application of AI in medical fields. Since ChatGPT demonstrated improved handling of professional knowledge in our study at re-prompting, it may be educationally useful, and our results also suggest the need for repeated training in medical knowledge when using ChatGPT models in the medical field.

Interestingly, some comparisons for accuracy between ChatGPT models (i.e., model 3.5 re-prompt and model 4.0 first prompt; 3.5 re-prompt and 4.0 re-prompt; 4.0 first prompt and 4.0 re-prompt) showed no statistical significance. Moreover, there was no statistical significance in the comparisons between the first prompt of model 3.5 and student results. These findings imply that, given the same question, the chances of getting a correct answer would be higher (vs. first prompt of model 3.5) in the re-prompt of model 3.5, the first prompt of model 4.0, and the re-prompt of model 4.0. Then, upon comparing student results with ChatGPT model results, we found the accuracy of the first prompt of model 3.5 to be the most similar to that of students.

When considering difficult questions, the accuracy of students and the first prompt of model 3.5 was similar, whereas model 4.0 and all the other prompts showed higher chances of providing correct answers (vs. students). When considering the easy questions, both ChatGPT models and all different model prompts showed a higher probability of providing correct answers (vs. students). We also checked whether ChatGPT model accuracy would vary by question difficulty, finding that the first prompt of model 3.5 underperformed in relation to other models, although not to a statistically significant extent. It is important to underline here the possibility of sample size issues, as our sample may have been insufficient for the analyses; if the sample size is sufficient, it may be that the easier the question, the higher the model accuracy. This is an examination to be conducted by future research.

There is also another concern reported about ChatGPT, as although it has been provided in numerous languages, it may be less fluent in languages other than English.^[21]^ This is mostly because LLM performance depends on the training data, and most of the training data available is in English. This makes it necessary to examine the effectiveness of ChatGPT in handling the Korean language, for which there is less training data available. In the current study, the questions in the examination were written in Korean and entered into ChatGPT without translation. After extracting the results, we translated the incorrect questions into English and asked them again to the ChatGPT models to explore the potential bias. The results for the English translated questions did not yield performance differences for ChatGPT. Still, for high-level tasks, the performance of ChatGPT is generally better in English than in other languages.^[21]^ We believe that the non-differential results between the English and Korean versions of the questions may be because our questions involved short answers, and the translated texts were not sufficiently complex to cause disagreements in interpretation with the original text. This hampers our ability to suggest the level of the processing capabilities of ChatGPT in the Korean language. Even if AI answered certain Korean items correctly, this does not necessarily guarantee accurate comprehension. Therefore, further research verifying natural language and multilingual processing is required.

The limitations of this study are as follows. First, the number of questions was relatively small, the student sample was not representative of all medical students in Korea, and the examination used was not a national board examination; these characteristics hinder the generalization of our findings. Therefore, the present findings may be constrained by factors such as the specificity of the content, the level of subject expertise, the limited number of items, and the sample size. In light of these considerations, conclusions regarding external validity should be interpreted with caution. Second, the questions were limited to a specific medical department, and this was mostly because of the limited capabilities of ChatGPT at the time of the study, which constrained us to comparing only specific department questions. The development of ChatGPT may allow future comparative studies based on examinations from additional medical departments. Third, the question containing media was excluded because the image-capable ChatGPT models were not publicly accessible at the time of this study. However, considering that the correct response rate for the 1 excluded question was 91% and that it was considered an easy question in the verification of a board-certified professor, it is unlikely that its exclusion affected the discriminatory power of the overall examination. Finally, we could not estimate the current level of ChatGPT, as we derived our results from data extracted in just one day in 2023. Current ChatGPT models may have been trained with more information than when we conducted this study, implying the possibility of different results. Researchers are invited to constantly examine the limitations and potential pitfalls of ChatGPT.

Despite these concerns, our findings show that ChatGPT may be a reasonable tool for providing medical information to answer medical questions similar to those in the examination explored in this study. This study is significant in several aspects: it is one of the few studies to apply the exact same exam questions that were administered to medical students directly to ChatGPT and additionally compare the performance across different versions of ChatGPT; few previous studies have evaluated ChatGPT using a regional language; and by administering the same test in both the regional language and English, this study revealed that there is no significant difference in ChatGPT’s analytical performance depending on the language, highlighting the model’s consistent reasoning ability regardless of linguistic context. Therefore, although this study has limited generalizability, it does deliver insights into this emerging AI tool.

## 5. Conclusion

The ChatGPT models, especially model 4.0, exhibited excellent performance and learning ability in the medical education field. Therefore, although ChatGPT may contain some incorrect information, it can be considered a reliable LLM tool. However, this must be constantly verified to ensure that it provides reliable information. Blindly trusting AI’s high accuracy can cause problems, especially in situations involving patient lives. Educators must be cautious when interpreting ChatGPT output data and applying it to clinical situations or educational purposes.

## Acknowledgments

The authors would like to thank the Medical Statistics Support Team, Research Institute of Healthcare Bigdata, Dankook University College of Medicine.

## Author contributions

**Conceptualization:** Kyung Wook Kim, Hong Bae Jeon, Dong Uk Lee, Bum-Jin Shim.

**Funding acquisition:** Kyung Wook Kim, Bum-Jin Shim.

**Project administration:** Kyung Wook Kim, Bum-Jin Shim.

**Supervision:** Kyung Wook Kim, Bum-Jin Shim.

**Formal analysis:** Hong Bae Jeon, Dong Uk Lee, Bum-Jin Shim.

**Resources:** Hong Bae Jeon, Yoon Sung Jeon.

**Software:** Hong Bae Jeon, Jong Hyuk Choi, Yoon Sung Jeon.

**Validation:** Hong Bae Jeon, Dong Uk Lee.

**Data curation:** Jong Hyuk Choi, Dong Uk Lee, Bum-Jin Shim.

**Investigation:** Jong Hyuk Choi, Dong Uk Lee.

**Visualization:** Jong Hyuk Choi, Yoon Sung Jeon.

**Methodology:** Dong Uk Lee.

**Writing – original draft:** Kyung Wook Kim, Jong Hyuk Choi, Bum-Jin Shim.

**Writing – review & editing:** Kyung Wook Kim, Bum-Jin Shim.
